# Mechanisms Underlying Hox-Mediated Transcriptional Outcomes

**DOI:** 10.3389/fcell.2021.787339

**Published:** 2021-11-16

**Authors:** Brittany Cain, Brian Gebelein

**Affiliations:** ^1^ Division of Developmental Biology, Cincinnati Children’s Hospital Medical Center, Cincinnati, OH, United States; ^2^ Department of Pediatrics, University of Cincinnati College of Medicine, Cincinnati, OH, United States

**Keywords:** Hox, transcription factor, chromatin accessibility, *cis*-regulatory modules (CRMs), protein-protein interaction

## Abstract

Metazoans differentially express multiple Hox transcription factors to specify diverse cell fates along the developing anterior-posterior axis. Two challenges arise when trying to understand how the Hox transcription factors regulate the required target genes for morphogenesis: First, how does each Hox factor differ from one another to accurately activate and repress target genes required for the formation of distinct segment and regional identities? Second, how can a Hox factor that is broadly expressed in many tissues within a segment impact the development of specific organs by regulating target genes in a cell type-specific manner? In this review, we highlight how recent genomic, interactome, and *cis*-regulatory studies are providing new insights into answering these two questions. Collectively, these studies suggest that Hox factors may differentially modify the chromatin of gene targets as well as utilize numerous interactions with additional co-activators, co-repressors, and sequence-specific transcription factors to achieve accurate segment and cell type-specific transcriptional outcomes.

## Introduction

Hox genes have long fascinated developmental biologists for the essential roles that they play in specifying different segment and regional identities along the developing anterior-posterior (A-P) axis of metazoans. Classic genetic studies first revealed that Hox gene mutations can result in homeotic transformations, and thereby cause one part of the organism to be transformed into the likeness of another region. As an example, *Drosophila* with Hox mutations can have obvious developmental abnormalities that include the misspecification of appendages as evidenced by the transformation of antennae into legs (Kaufman et al., 1980; Abbott and Kaufman, 1986; Schneuwly et al., 1987; Casares and Mann, 1998) or the conversion of the haltere into an extra set of wings (Lewis, 1978; Bender et al., 1983; Carroll et al., 1995). Subsequent studies in other organisms including a variety of vertebrate animals revealed that mutations within the highly conserved Hox gene family can cause a wide variety of homeotic transformations across metazoans as reviewed by Mark et al. (1997) and Quinonez and Innis (2014).

Hox genes were originally discovered in *Drosophila melanogaster.* In total, *Drosophila* has eight Hox genes that are separated into two distinct chromosomal clusters: The *Antennapedia* cluster consists of five Hox genes [*labial* (*lab*), *proboscipedia* (*pb*), *Deformed* (*Dfd*), *Sex combs reduced* (*Scr*), and *Antennapedia* (*Antp*)] that collectively regulate head and anterior thoracic development, whereas the three Hox genes in the *Bithorax* cluster [*Ultrabithorax* (*Ubx*), *abdominal-A* (*abd-A*), and *Abdominal-B* (*Abd-B*)] specify cell fates within the third thoracic segment and the abdominal segments (Morata et al., 1990; Maeda and Karch, 2009). In general, the order of the Hox genes on the chromosome correspond with the location along the A-P axis that the Hox genes act in the embryo (Lewis, 1978; Mann, 1997; Noordermeer and Duboule, 2013; Luo et al., 2019; Hajirnis and Mishra, 2021). For example, genes at the 3′ end of the Hox gene cluster tend to mediate anterior development whereas the 5′ genes tend to control posterior structures. In contrast to the single set of eight Hox genes in *Drosophila*, vertebrates have undergone genome duplication events such that humans have four distinct Hox clusters (labeled HOXA, HOXB, HOXC, and HOXD, respectively) encoding 39 Hox genes that have been categorized into 13 paralogs (HOX1-13). Importantly, the mammalian Hox genes exhibit the same spatial collinearity along the A-P axis as in *Drosophila* (Duboule and Dolle, 1989; Graham et al., 1989). For example, HOX1 genes on the 3′ end of each cluster regulate anterior structures including the hindbrain (Singh et al., 2020), while HOX13 genes on the 5’ end of each cluster control posterior and distal structures including digit development (Desanlis et al., 2020). Based on sequence conservation, the relative positions of each Hox gene within a cluster, and their roles in A-P patterning, the Hox genes have been broadly categorized into anterior (*lab*, *pb*, *Dfd*, and *Scr* in *Drosophila* and *Hox1-5* in vertebrates), central (*Antp*, *Ubx*, and *abd-A* in *Drosophila* and *Hox6-8* in vertebrates), and posterior groups (*Abd-B* in *Drosophila* and *Hox9-13* in vertebrates) (Hueber et al., 2010). It is important to note that not all Hox paralogs remain in each of the duplicated vertebrate Hox clusters. For example, cluster HOXB does not have posterior factors HOXB10-B12, and cluster HOXC lacks paralogs of HOXC1-C3 in humans (Mark et al., 1997). In short, metazoans encode variable numbers of Hox genes that are typically found clustered along the chromosome to specify the different cell fates that form along the A-P axis body plan.

The mysteries underlying how Hox genes control distinct body regions only grew upon the discovery that each encodes a homeodomain transcription factor (TF) capable of binding highly similar AT-rich DNA sequences (McGinnis et al., 1984a; McGinnis et al., 1984b). In fact, Hox genes are members of a much larger homeodomain TF family that consists of over 200 members in mammals, and many of these genes control distinct developmental processes and fates despite encoding TFs that bind highly similar DNA sequences (Berger et al., 2008; Jolma et al., 2013; Bürglin and Affolter, 2016). Taken together, these conflicting genetic and biochemical findings raise a fundamental paradox: How can a family of homeodomain TFs capable of binding highly similar DNA sequences *in vitro*, regulate distinct and diverse cell fates *in vivo*?

During the past two decades, many molecular, genetic, and genomic approaches have begun to reveal that numerous mechanisms likely underlie the ability of Hox TFs to specify different cell fates along the A-P body axis. In total, these studies have made considerable progress in defining mechanisms that enhance Hox DNA target specificity, especially by the formation of larger DNA binding complexes with other TFs. For example, the Extradenticle (Exd, *Drosophila*)/Pre-B cell leukemia homeobox (Pbx, vertebrate) and/or Homothorax (Hth, *Drosophila*)/Myeloid ecotropic viral integration site (Meis, vertebrate) TFs have been shown to form cooperative DNA binding complexes with Hox TFs and thereby enhance Hox DNA binding specificity (Mann and Affolter, 1998; Moens and Selleri, 2006; Merabet and Mann, 2016). Through a combination of structural, biochemical, and genetic studies, the formation of Hox/Exd and Hox/Pbx complexes have uncovered several key concepts that underlie how Hox TFs gain DNA binding specificity including the critical role of not just nucleotide identity but DNA shape (Zeiske et al., 2018), the concept of latent specificity (Slattery et al., 2011), and the importance of low affinity versus high affinity binding sites (Crocker et al., 2015; Zandvakili et al., 2019). These mechanisms, which by and large are used to increase Hox target gene specificity, have been reviewed in several excellent articles (Merabet and Mann, 2016; Kribelbauer et al., 2019; De Kumar and Darland, 2021).

In this review, we focus on how large-scale genomic and interactome data have uncovered numerous potential Hox regulatory elements and protein interactors that present both new opportunities and challenges. Genomic DNA binding studies from tissues and cells have revealed that Hox TFs, like most sequence-specific TFs, bind thousands of potential *cis*-regulatory elements but only a subset of these binding events are likely to be associated with significant changes in the expression of nearby genes (Walter et al., 1994; Farnham, 2009; Biggin, 2011; Choo and Russell, 2011; Walhout, 2011; Fisher et al., 2012). In addition, comparative studies between Hox TFs have revealed differences in their ability to bind inaccessible (i.e., closed chromatin) DNA elements. Such differences in ability to bind DNA wrapped in nucleosomes may indicate that Hox TFs have the potential to elicit pioneer-like activities that promote the opening of closed chromatin, thereby expanding the already large number of possible genomic binding sites. However, since Hox TFs are capable of mediating both transcriptional activation and repression, simply detecting Hox TF binding to an element cannot easily be used to predict transcriptional outcome. Intriguingly, protein-protein interaction assays have uncovered that Hox TFs can interact with many different proteins including other sequence-specific TFs as well as factors involved in mediating gene activation and repression. Integrating these large-scale findings with existing *cis*-regulatory logic studies of confirmed Hox target genes suggests that Hox TFs are likely to require numerous protein-protein interactions with other TFs to gain the required regulatory specificity to ensure accurate gene activation or repression outcomes occur in a reproducible and robust manner.

## Hox Transcription Factor Binding and Chromatin Accessibility

Hox factors, like all TFs, must bind specific DNA regulatory elements to mediate accurate transcriptional responses. Since all the cells within an organism have the same genomic material, differences in the chromatin landscape of a cell can play a large role in dictating which DNA elements are available for transcription factor binding. Thus, chromatin accessibility helps to define which genes can be activated during the specification of distinct cell fates along the body plan. Intriguingly, comparative genomic accessibility studies using Formaldehyde-Assisted Isolation of Regulatory Elements sequencing (FAIRE-seq) on *Drosophila* imaginal discs revealed that the wing, haltere, and metathoracic leg imaginal discs have very similar chromatin profiles (McKay and Lieb, 2013). For example, comparison between the wing and haltere imaginal discs showed that except for genomic regions flanking the *Ultrabithorax* (*Ubx*) Hox gene these two tissues have largely identical accessible *cis* regulatory elements (McKay and Lieb, 2013). Similar results were obtained using the Assay for Transposase-Accessible Chromatin sequencing (ATAC-seq) methods with ∼98% of the accessible DNA sequences being the same between age-matched wing and haltere discs (Loker et al., 2021).

The above findings suggest that the Ubx Hox factor, which is differentially expressed in the *Drosophila* imaginal discs, directs the formation of different cell and tissue fates by regulating distinct target genes within highly similar chromatin landscapes. But does the expression of this Hox TF alter the chromatin landscape during the process of cell fate specification and morphogenesis? A recent elegant study addressed this question to better define the role of the Ubx TF in regulating haltere development by focusing on the relatively small percentage of loci (∼2% of accessible regions) that were differentially accessible in haltere versus wing discs (Loker et al., 2021). Importantly, Loker et al. combined chromatin accessibility data with Ubx Chromatin Immunoprecipitation sequencing (ChIP-seq) assays and transcriptomics (RNA-seq) to show that Ubx genomic binding correlates with the opening and closing of specific loci to mediate distinct transcriptional outputs during *Drosophila* haltere development (Loker et al., 2021). In particular, they found that Ubx could modify the chromatin landscape to both reduce chromatin accessibility to repress gene transcription in the capitulum and proximal hinge of the haltere and increase chromatin accessibility to activate gene transcription in the distal hinge with the aid of the Hth and Exd Hox co-factor proteins (Loker et al., 2021). Since *Ubx* is required for haltere fate and the loss of *Ubx* function results in the transformation of haltere tissue into wing tissue (Lewis, 1978; Bender et al., 1983; Carroll et al., 1995), these data are congruent with the idea that the primary difference between these two serially homologous appendages is the expression of *Ubx* and that once expressed, the Ubx TF directs haltere development by modulating chromatin accessibility and target gene expression within an initial chromatin landscape capable of forming either a wing or a haltere (McKay and Lieb, 2013; Loker et al., 2021). Thus, while many of the accessible genomic regions across imaginal disc tissues are the same, Hox TFs are likely to modify this landscape to activate and/or repress key target genes during cell fate specification and morphogenesis.

**FIGURE 1:**
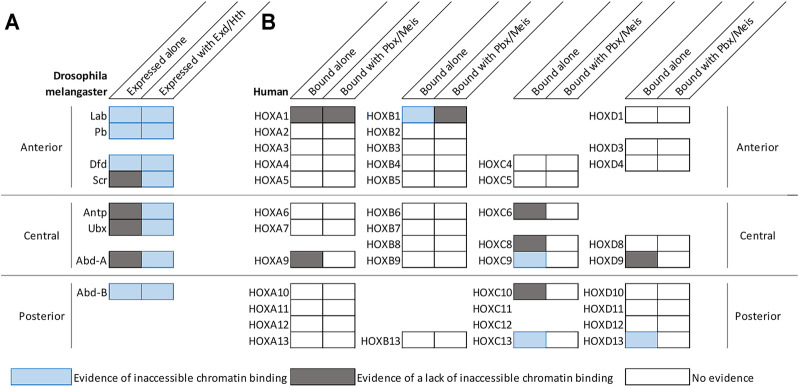
Select Hox factors can bind inaccessible chromatin with and/or without the help of common co-factors. **(A)** Diagram summarizing the genomic DNA binding activities of Hox TFs in *Drosophila* Kc167 cells (data from Beh et al., 2016; Porcelli et al., 2019). By comparing the chromatin accessibility profiles of cells prior to Hox factor transfection and genome binding profiles after Hox factor transient transfection in Kc167 cells, Beh et al. and Porcelli et al. demonstrated that anterior factors and posterior *Drosophila* factors tend to have the ability to bind inaccessible chromatin (Beh et al., 2016; Porcelli et al., 2019). Furthermore, Exd and Hth expression tend to enhance a factor’s ability to bind to inaccessible chromatin (Beh et al., 2016; Porcelli et al., 2019). It is important to note that the ability to bind inaccessible chromatin of Abd-B was not enhanced and the ability of Scr was only slightly enhanced by Exd/Hth. **(B)** Diagram summarizing the genomic DNA binding activities of human Hox factors in motor neuron cells (Bulajić et al., 2020), mouse embryonic stem cells (Singh et al., 2021), and mouse limb buds (Desanlis et al., 2020). The genomic binding and accessibility profiles were intersected to assess inaccessible chromatin binding. Nearby PBX and MEIS motifs were used to determine co-binding. *Drosophila* and human Hox factors follow a similar trend that posterior factors can bind inaccessible chromatin more so than central factors.

**FIGURE 2:**
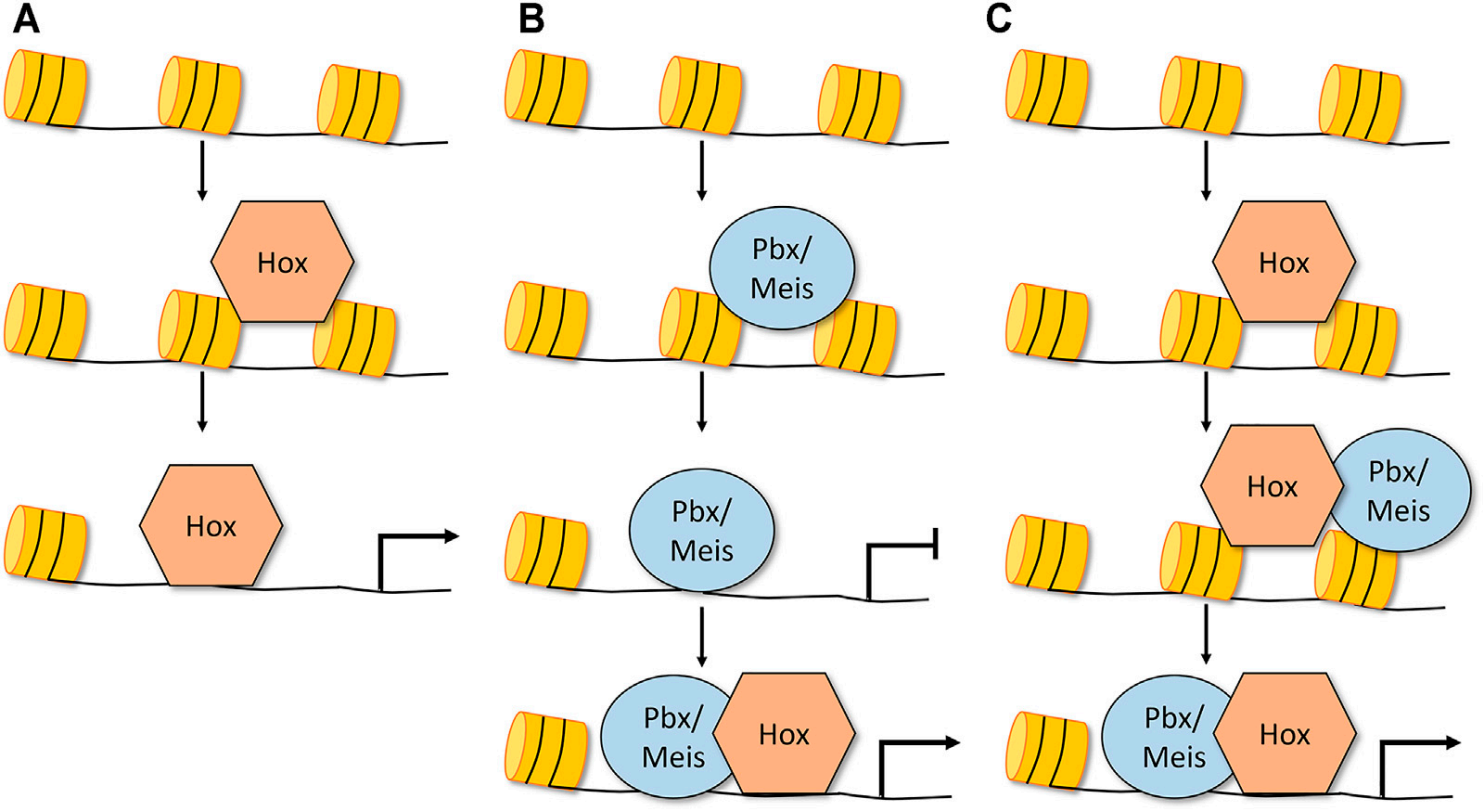
Mechanisms by which Hox factors could exhibit pioneer-like activity and alter chromatin accessibility. **(A)** Dfd and Abd-B Hox factors alone can bind to inaccessible chromatin and increase the chromatin accessibility of its targets without co-factor expression (Porcelli et al., 2019). **(B)** Pbx/Meis in vertebrates have been shown to bind inaccessible chromatin and promote chromatin opening. In this model, the Hox factor gains access to accessible DNA, forms a complex with Pbx/Meis, and this larger TF complex is required for accurate target regulation (Sagerstrom, 2004; Choe et al., 2014; Mariani et al., 2021). **(C)** Another potential model is that Hox TFs, a subset of which are capable of binding inaccessible DNA, recruit Exd/Pbx and Hth/Meis and together these complexes promote chromatin opening (Porcelli et al., 2019). Note that in each of these models, the role of the Hox factor in chromatin opening has not yet been confirmed.

The finding that Hox TF binding can increase genomic accessibility raises the possibility that Hox TFs have pioneer-like activities. By definition, pioneer transcription factors can both bind DNA that is wrapped around a nucleosome and promote chromatin remodeling to make DNA elements accessible for other TFs (Iwafuchi-Doi and Zaret, 2014; Zaret, 2020). To assess the ability of Hox TFs to bind inaccessible DNA and promote chromatin opening, recent comparative genomic binding and accessibility studies have been performed for Hox TFs in both a *Drosophila* cell line (Kc167 cells) (Beh et al., 2016; Porcelli et al., 2019) and in a mouse motor neuron progenitor culture system (Bulajić et al., 2020). Intriguingly, these data indicate that some, but not all, Hox factors can readily bind inaccessible chromatin. By intersecting ATAC-seq and ChIP-seq profiles of the eight *Drosophila* Hox TFs, Porcelli et al. showed that this ability to bind inaccessible chromatin is shared by the anterior factors, Lab, Dfd, and Pb, as well as the posterior Hox factor, Abd-B (Figure 1A; Porcelli et al., 2019). Further, by comparing ATAC-seq profiles before and after inducing Dfd and Abd-B expression in respective Kc167 cell lines, Porcelli et al. found that Dfd and Abd-B can increase chromatin accessibility of their targets (Porcelli et al., 2019). These findings are consistent with a previous finding that 42% of Abd-B specific peaks were bound outside of the cells DNaseI accessible regions in Kc167 cells (Beh et al., 2016). The enhanced ability of Abd-B to bind inaccessible chromatin was also supported by studies of the mammalian Abd-B orthologs in neural progenitors and undifferentiated motor neurons (Bulajić et al., 2020). In particular, Bulajić et al. found that the HOXC9 and HOXC13 posterior Hox TFs bound significantly more inaccessible genomic regions than the HOXC6 and HOXC8, which are classified as central Hox TFs (Figure 1B; Bulajić et al., 2020). Consistent with these findings, the HOXD13 TF also demonstrated pioneer factor-like activity by increasing chromatin accessibility of targets to guide proximal to distal limb development (Figure 1B; Desanlis et al., 2020), supporting a mechanism in which select Hox factors can bind inaccessible chromatin and increase chromatin accessibility of its targets (Figure 1; Figure 2A).

While the above findings are congruent with the idea of the posterior Abd-B-like Hox factors being able to readily bind inaccessible DNA, additional studies revealed that not all posterior Hox TFs may equally share such properties. For example, comparative studies between several posterior HOX TFs in the motor neuron progenitor assay revealed clear differences with HOXC9 and HOXC13 binding many more inaccessible regions than HOXC10, HOXA9, or HOXD9 (Figure 1B; Bulajić et al., 2020). Moreover, the ability of the human HOXC13 factor to bind inaccessible DNA was predominantly influenced by the DNA binding domain and C-terminus (Bulajić et al., 2020). Thus, while it has been argued based on structural studies that posterior Hox TFs may have enhanced binding to inaccessible DNA due to high affinity electrostatic interactions between the narrow groove of DNA and the Hox N-terminal arm of the homeodomain (LaRonde-LeBlanc and Wolberger, 2003; Beh et al., 2016), we currently lack a molecular understanding of why only a subset of posterior HOX TFs readily bind inaccessible chromatin.

## The Impact of Pbx/Exd and Meis/Hth on Altering Hox Binding in Chromatin

Hox factors are well known to interact with the Pbx/Exd and Meis/Hth TFs to enhance DNA binding specificity on naked DNA *in vitro* (Mann and Affolter, 1998; Uhl et al., 2010; Merabet and Mann, 2016). More recently, these factors have also been shown to influence the binding of Hox TFs as well as other TFs to genomic DNA elements embedded in chromatin. The first study describing such an activity for Pbx and Meis was in association with MyoD, a non-Hox basic-Helix-Loop-Helix transcription factor that promotes muscle cell development (Berkes et al., 2004). Pbx was shown to bind the inactive *myogenin* promoter in undifferentiated C2C12 myoblast cells at a time-point that preceded MyoD binding by 6 h (Bergstrom et al., 2002; Berkes et al., 2004). The subsequent binding of MyoD with Pbx during differentiation correlated with *myogenin* promoter activation, consistent with previous studies that found MyoD is able to remodel chromatin and activate target genes (Gerber et al., 1997). Together, these findings suggest that Pbx is binding to and marking the inaccessible chromatin for activation upon recruitment of MyoD, a mechanism that might extend to Pbx’s interactions with Hox factors (Sagerstrom, 2004). In fact, Choe et al. found that Pbx/Meis bound numerous loci as early as the zebrafish blastula and that later in development the Hoxb1a TF was required for these loci to become fully active (Choe et al., 2014). More recently, Mariani et al. used a combination of DNA accessibility assays, Pbx ChIP-seq assays, and transcriptomics on wild type and Pbx knockout cells undergoing paraxial mesoderm differentiation to show that Pbx factors are required to bind and open essential chromatin regions during the maturation of paraxial mesoderm cells (Mariani et al., 2021). Importantly, the authors used genome editing to show that a Pbx binding site in a regulatory element of the *msgn1* gene that specifies paraxial mesoderm cell fate is required for its chromatin accessibility. Thus, either the loss of the Pbx protein or the disruption of the Pbx binding site resulted in the loss of *msgn1* enhancer DNA accessibility and *msgn1* gene activation (Mariani et al., 2021). These data support a model in which Pbx marks and opens the inaccessible chromatin for subsequent gene regulation by Hox factors (Figure 2B).

In *Drosophila*, Porcelli et al. systematically assessed how the Exd and Hth co-factors impact genomic accessibility and Hox DNA binding profiles by taking advantage of the fact that Kc167 cells lack Hth expression, which thereby restricts Exd to the cytoplasm (Porcelli et al., 2019). These studies revealed that the expression of Hth, which concomitantly localizes Exd to the nucleus, generally increased the genomic binding of all the *Drosophila* Hox factors but Abd-B to inaccessible chromatin (Figure 1A; Porcelli et al., 2019). This was previously shown for Ubx in which ∼30% of the Ubx and Hth specific binding sites did not intersect with the cell line’s DNase1 profile prior to Hox gene expression, whereas in the absence of Hth and nuclear Exd only ∼5% of Ubx bound regions intersected with this DNaseI inaccessible chromatin profile (Beh et al., 2016). Moreover, by comparing chromatin profiles before and after Ubx and Hth induction, Ubx was shown to open the surrounding chromatin of its targets with the help of Hth (Porcelli et al., 2019). These data support the idea that the formation of Ubx/Hth/Exd complexes can promote chromatin remodeling and DNA accessibility, which is consistent with the findings that Ubx increases chromatin accessibility to activate gene transcription in the Hth and Exd expressing cells of the distal hinge in the haltere disc (Loker et al., 2021).

In agreement with these *Drosophila* findings, a recent study in mouse embryonic stem cells studies found that like Lab (Porcelli et al., 2019), the HOXB1 homologue is capable of binding to both inaccessible and accessible DNA (Singh et al., 2021). Intriguingly, by also performing ChIP-seq assays for PBX1 and various chromatin marks, the authors found that those HOXB1 peaks found in inaccessible DNA were not bound by PBX1 and were predominantly located in gene deserts of nucleosome-bound chromatin. In contrast, the HOXB1 regions that were also bound by PBX1 tended to be in more accessible chromatin that correlated with open chromatin marks such as H2K27ac, H3Kme1, and H3Kme3 (Figure 1B; Singh et al., 2021). These findings suggest that while HOXB1 has the capacity to bind inaccessible DNA, it may have a limited ability to convert that binding event into accessible chromatin unless co-bound with the PBX1 factor.

Collectively, the above genomic data in both *Drosophila* and vertebrates support the idea that the Pbx/Exd and Meis/Hth factors have some degree of pioneer TF activity. Consistent with this model, a recent nucleosome consecutive affinity purification-systematic evolution of ligands by exponential enrichment assay provided evidence that MEIS3 is capable of binding nucleosome bound DNA *in vitro* (Zhu et al., 2018), and a comparative study of pioneer TFs highlighted that PBX contains a truncated alpha recognition helix that mimics the structure that allows the FOXA3, OCT4, PU1, and ASCL1 pioneer TFs to bind nucleosome bound DNA (Fernandez Garcia et al., 2019). In total, these studies provide support for the following model: the Pbx/Exd and Meis/Hth factors can bind inaccessible DNA, promote chromatin opening, and ultimately regulate target gene expression *via* the subsequent recruitment of Hox TFs as well as other non-Hox TFs such as MyoD (Figure 2B). What remains less clear is if the Hox TFs are only involved in the final step of target gene activation or if the Hox TFs also participate with Pbx/Exd and Meis/Hth in the process of chromatin remodeling. Moreover, since at least a subset of Hox TFs also bind inaccessible DNA, it is possible that at some regulatory elements Hox TFs can use a pioneer-like activity to bind inaccessible DNA and subsequently recruit the Pbx/Exd and/or Meis/Hth factors to open chromatin and regulate target gene expression (Figure 2C). Thus, the differential ability of Hox TFs and the Pbx/Exd and Meis/Hth TFs to bind accessible versus inaccessible DNA provide an additional potential regulatory mechanism that may underlie how the anterior, central, and posterior Hox TFs accurately control target gene expression during animal development.

## Hox Factors as Multi-Functional Transcriptional Activators and Repressors

Once bound to DNA, Hox factors ultimately function by altering the expression of downstream target genes. Unlike some TFs that are thought to function predominantly as transcriptional activators or repressors, the Hox TFs are capable of mediating both transcriptional outcomes (Pearson et al., 2005; Zandvakili and Gebelein, 2016). In the past, considerable work has been done to map activation and repression domains of the Hox factors as well as to determine how mutating these regions impacts transcriptional output. For example, a structure function study of Abd-A in *Drosophila* revealed how a Hox protein can utilize multiple distinct Exd interaction domains to differentially regulate target genes and morphological outcomes (Merabet et al., 2011). Further, a combination of mutational analyses and transcriptional output assays using Gal4 drivers showed that Dfd in *Drosophila* (Li et al., 1999) and HoxA7 in NIH3T3 cells (Schnabel and Abate-Shen, 1996) as well as HoxD4 in P19 embryonal carcinoma cells (Rambaldi et al., 1994) possessed a proline alanine rich region in the N-terminus that can activate transcriptional output. However, this activity was masked by the homeodomain and C-terminus in the context of the full proteins. In fact, there is increasing evidence that the homeodomain itself can be a large driver of transcriptional repression, and that the extent of this repression is paralog specific. A recent study quantitively measured protein domain transcriptional activity using a novel high-throughput sequencing technique, HT-recruit (Tycko et al., 2020). This study fused a large library of TF protein domains to the rTetR DNA binding domain within a lentivirus and assessed their ability to alter a citrine reporter gene under the control of TetO binding sites. After subjecting infected cells to doxycycline, cells were sorted for citrine-ON versus citrine-OFF and the read count ratio between the off and on cells was used to quantify the repression capability of each protein domain. Through this technique, they discovered that the repression capability of the Hox homeodomains was colinear and correlated with paralog such that posterior Hox factors had a more potent repression activity than the anterior Hox factors (Tycko et al., 2020). The authors then connected the enhanced repression of posterior factors to a more positively charged N-terminal arm in the homeodomain, specifically a RKKR motif (Tycko et al., 2020). This connection is consistent with a previous mutational study that found that mutating a similar region of HoxA7 to the amino acids of HoxB4 resulted in reduced repression activity (Schnabel and Abate-Shen, 1996). Altogether, these findings highlight the importance of the homeodomain in repression as well as exemplifies how transcriptional outputs across Hox proteins can vary. Moreover, these data provide further evidence that the same Hox TFs, such as HoxA7, HoxD4, and Abd-A, can have both functional activation and repression domains.

Given that Hox TFs have the capacity to both activate and repress transcription, it is not surprising that a wide variety of co-activator and co-repressor proteins have been shown to physically interact with Hox TFs as reviewed in (Mann et al., 2009; Zandvakili and Gebelein, 2016; De Kumar and Darland, 2021). Many large scale interactome analyses have been performed with Hox factors, and each of these have identified a substantial number of potential protein-protein interactions that could modify the ability of the Hox TFs to mediate gene activation and/or gene repression (Giot et al., 2003; Stanyon et al., 2004; Rual et al., 2005; Yu et al., 2011; Lambert et al., 2012; Rolland et al., 2014; Bischof et al., 2018; Shokri et al., 2019; Carnesecchi et al., 2020; Luck et al., 2020). For example, the Ubx and Abd-A Hox factors were screened for interactions against 260 different TFs in the *Drosophila* embryo using a split-fluorescence assay coupled with ectopic expression using the Gal4-UAS system (Bischof et al., 2018). Unexpectedly, both of these Hox TFs interacted with a large number of the tested TFs, as Ubx interacted with 163 of the 260 TFs (62%), and Abd-A interacted with 149 of the TFs (57%) (Bischof et al., 2018). However, it should be noted that an additional large-scale TF-TF interaction screen tested a number of different Hox TFs using a yeast 2-hybrid assay and found that the Hox TFs, including Ubx and Abd-A, interact with relatively few tested TFs (Shokri et al., 2019). These conflicting results are likely to be attributed to differences in sensitivity between the two assays as well as the fact that the fluorescence complementation assay was performed in *Drosophila* cells that express additional co-factor proteins that may allow large scale complex formation whereas the two-hybrid approach was performed in yeast. More recently, a proximity-dependent Biotin IDentification (BioID) assay in multiple cell types of the *Drosophila* embryo revealed that Ubx interacts with many proteins involved in processes from chromatin modification to mRNA processing (Carnesecchi et al., 2020). Surprisingly, however, while most of the BioID identified Ubx interactors were found to occur in a tissue-specific manner, the vast majority of the proteins that do interact with Ubx are broadly expressed across many tissues (Carnesecchi et al., 2020). This finding raises the possibility that the ability of Ubx to interact with ubiquitous regulatory proteins can be modified in a tissue-specific manner, although the mechanisms regulating such tissue-specific interactions are currently unknown. Nevertheless, these data raise the possibility that the Hox TFs gain in DNA binding specificity by forming complexes with numerous additional TFs, many of which are expressed in a tissue-restricted manner, and gain in regulatory specificity (i.e., activate versus repress) by interacting with many different co-activator and co-repressor proteins that are widely expressed in numerous cell types.

## Case Studies on the *cis*-Regulatory Logic of Hox Transcription Factors

To better understand how Hox TFs regulate target genes in specific tissues, a select number of *cis*-regulatory modules (CRMs) have been extensively characterized using a combination of DNA binding assays, transcriptional reporter assays, and loss- and gain-of-function genetics. In this review, we are going to focus on our current knowledge of the *cis*-regulatory logic of two well-characterized *Drosophila* CRMs, one of which is specifically regulated by Abd-A and the other is regulated by the Abd-A, Ubx, and Antp Hox factors. Intriguingly, Abd-A regulates these two CRMs in different cell types and in opposing ways. In the developing peripheral nervous system, Abd-A triggers the secretion of epidermal growth factor ligands from a specific subset of abdominal sensory organ precursor cells by activating the expression of the *rhomboid* (*rho*) serine protease gene *via* a highly conserved CRM called *RhoA* (Brodu et al., 2002; Li-Kroeger et al., 2008). In contrast, Abd-A, as well as Ubx, suppresses leg development in abdominal segments by repressing the expression of the *Distal-less* (*Dll*) homeodomain protein in ectodermal cells *via* the *Dll* conserved regulatory element (*DCRE*) (Vachon et al., 1992; Gebelein et al., 2002, Gebelein et al., 2004). In addition to being repressed by both Abd-A and Ubx in the abdomen, the *DCRE* CRM can also be activated by the Antennapedia (Antp) Hox factor in thoracic segments (Uhl et al., 2016).

**FIGURE 3:**
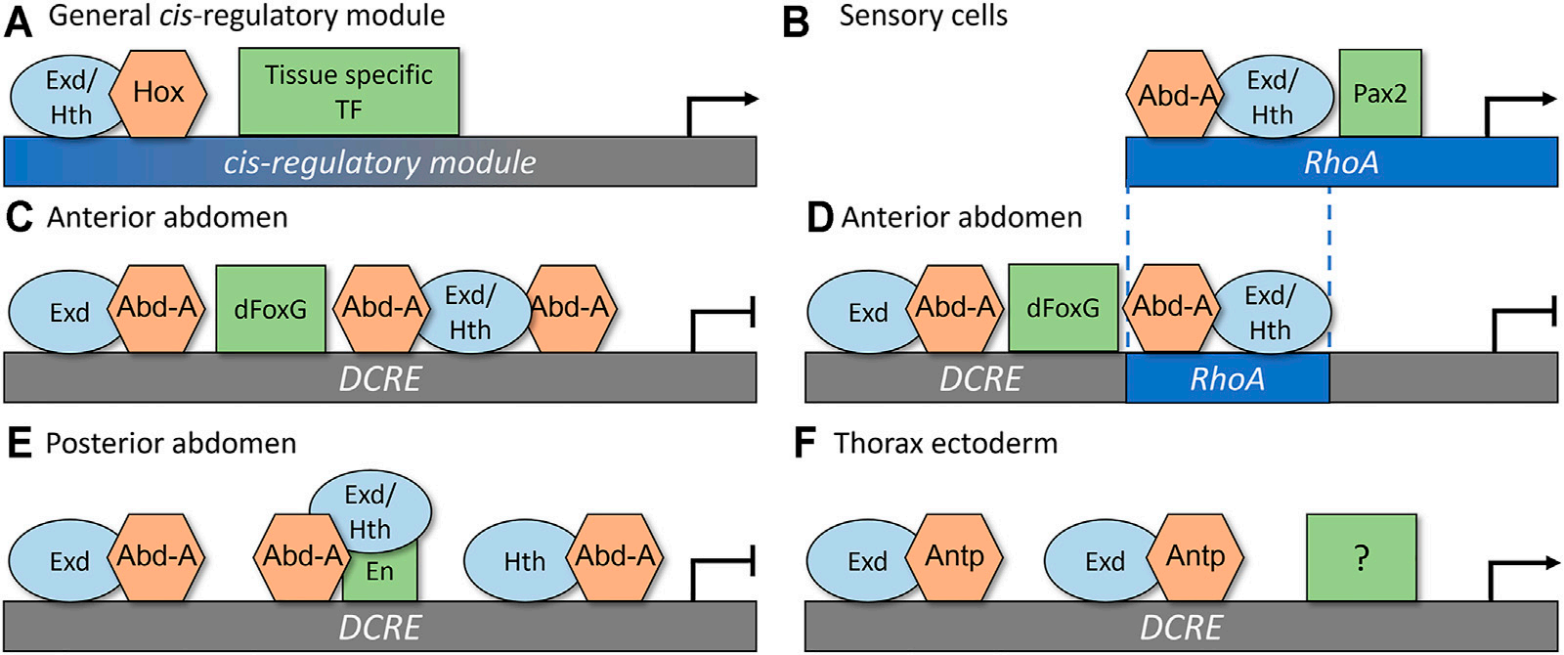
*Rhomboid-A* (*RhoA*) and *Distal-less conserved regulatory element* (*DCRE*) are regulated through tissue specific co-activators and co-repressors to mediate distinct transcriptional outputs. **(A)** A simplistic general model for how a Hox CRM yields cell-specific outputs *via* the direct integration of a Hox factor with the common co-factors Exd and Hth, and a tissue specific TF to mediate either activation or repression **(B)** In sensory cells, Abd-A, Exd, Hth, and Pax2 interact together to activate gene expression by forming complexes on the *RhoA* CRM. **(C)** In the anterior abdomen, Abd-A/Exd/Hth complexes bind together with dFoxG to repress the *DCRE* CRM. **(D)** Replacing two of the Hox binding sites from the *DCRE* with the Abd-A/Hth/Exd sites that mediate activation *via* the *RhoA* CRM did not alter the transcriptional response of the *DCRE*. These data show that the configuration of Hox binding sites does not dictate gene activation versus repression. Instead, it is the presence of nearby dFoxG sites [repression in **(D)**] or Pax2 sites [activation in **(B)**] that dictates transcriptional outcomes. **(E)** In the posterior abdomen, En interacts with Abd-A and Exd/Hth to repress the *DCRE* CRM. **(F)** In the thorax ectoderm, the Antp Hox factor cooperatively binds with Exd to stimulate gene expression. This activation activity does not require the dFoxG nor the En sites. In this model, we propose that an unknown tissue specific TF mediates activation of *DCRE* CRM to result in ectoderm specific gene activation.

To determine how these CRMs recruit specific Hox factors to mediate distinct cell type- and segment-specific outputs, comparative studies on the TF binding sites (TFBSs), the TF complexes, and the genetic requirements of each TF have revealed several insights into the principals underlying Hox *cis*-regulatory logic (Figure 3). First, the same Hox, Exd, and Hth binding sites are capable of mediating either activation or repression. The *RhoA* CRM contains a single set of adjacent Exd/Hth/Hox binding sites (Figure 3B; Brodu et al., 2002; Li-Kroeger et al., 2008), whereas the *DCRE* CRM contains three Hox sites, each of which is directly adjacent to a Exd or Hth site (Figures 3C,E; Gebelein et al., 2002, Gebelein et al., 2004; Uhl et al., 2016). Each configuration of binding sites is capable of cooperatively binding Abd-A/Hth/Exd complexes. Further, mutations within these binding sites disrupt the ability of Abd-A to either activate gene expression in sensory cells (Figure 3B; Li-Kroeger et al., 2012) or repress gene expression in the abdominal ectoderm (Figures 3C,E; Gebelein et al., 2002, 2004; Uhl et al., 2016). Moreover, swapping the “activating” Exd/Hth/Hox sites from the *RhoA* CRM into the *DCRE* demonstrated that the Abd-A Hox factor can also use this same configuration of binding sites to mediate transcriptional repression (Figure 3D; Zandvakili et al., 2019). These data suggest that differences in the conformation of Exd, Hth, and Hox TFBSs do not reveal how the Abd-A Hox complex mediates distinct outcomes in different cell types.

Second, accurate Hox-dependent transcriptional outcomes by the *RhoA* and *DCRE* CRMs require nearby TFBSs for additional tissue-restricted TFs (Figure 3A). For example, the *RhoA* CRM encodes a binding site for the Pax2 TF (Figure 3B, the *Drosophila* Pax2 gene name is *shaven*, *sv*, but for simplicity we will call it Pax2) and mutations within the *RhoA* CRM that disrupt Pax2 binding compromise Abd-A mediated activation (Li-Kroeger et al., 2012; Zandvakili et al., 2018). Moreover, Pax2, Abd-A, Exd, and Hth could utilize these TFBSs to form specific TF complexes on the *RhoA* CRM (Li-Kroeger et al., 2012) and altering the spacing and orientation between the Pax2 and Exd/Hth/Hox site disrupted *RhoA* activity in abdominal sensory organ cells (Zandvakili et al., 2019). Given that the expression of the *Drosophila Pax2* gene is predominately restricted to sensory organ cells in the embryo (Li-Kroeger et al., 2012), the direct regulation of the *RhoA* CRM by Abd-A and Pax2 provides insight into both the abdominal and sensory specific activity of this enhancer.

The *DCRE* CRM similarly requires additional TFBSs to mediate abdominal-specific repression by the Ubx and Abd-A Hox factors (Figures 3C,E; Gebelein et al., 2004). In fact, these two Hox factors were found to require different TFs in the anterior versus the posterior compartments of the abdominal segments. In the posterior compartment cells, the Ubx and Abd-A Hox factors form cooperative complexes with the Engrailed (En) TFs on adjacent binding sites within the *DCRE* (Figure 3E). In the anterior compartment, the Ubx and Abd-A Hox factors cooperate with the *Drosophila* FoxG factors, which are encoded by the largely redundant *sloppy-paired 1* (*slp1*) and *sloppy-paired 2* (*slp2*) genes *via* nearby Hox and dFoxG binding sites within the *DCRE* (Figure 3C; Gebelein et al., 2004). Moreover, like in the *RhoA* CRM, the spacing between the Hox and dFoxG TFBSs contributed to optimal activity as adding a 5 bp sequence disrupted repression (Zandvakili et al., 2019). However, adding longer space sequences between the Hox and dFoxG sites (+10, +15, and +20 bps) resulted in strong transcriptional repression, suggesting that unlike the Pax2 and Hox sites in *RhoA*, the configurations of dFoxG and Hox sites in the *DCRE* are not rigidly fixed to mediate transcriptional repression (Zandvakili et al., 2019). Since the dFoxG factors are specifically expressed in the anterior compartment cells, whereas En is specifically expressed in the posterior compartment cells, these findings again highlight how a CRM can integrate both Hox TFs and tissue-restricted TFs to yield accurate abdominal-specific outcomes.

Third, the *DCRE* CRM can contribute to both Hox-mediated transcriptional repression in the abdomen and Hox-mediated transcriptional activation in the thorax. In addition to repressing *Dll* expression in the abdomen, the *DCRE* can also use a subset of the Hox TFBSs to stimulate gene expression in the thoracic leg primordia cells *via* the Antp Hox TF (Uhl et al., 2016). In particular, Antp can utilize the two Hox/Exd sites, but not the adjacent Hth/Hox site, to stimulate *DCRE*-mediated activation in thoracic cells (Figure 3F). However, unlike *DCRE* mediated repression, the dFoxG and the En binding sites are not required for this Hox-dependent activity, suggesting that Antp is likely to cooperate with other TFs to stimulate the *DCRE* (Figure 3F; Uhl et al., 2016). Additional *cis*-regulatory studies on the *six2* target gene in mammals have also revealed that the same Hox binding sites can be used to activate *six2 via* Hox11 factors in the kidney and repress *six2 via* Hoxa2 in the branchial arch and facial mesenchyme (Yallowitz et al., 2009). Thus, the same Hox binding sites can contribute to both activation and repression, but the appropriate transcriptional response will likely depend upon integrating distinct combinations of additional TFs.

While the thorough characterization of the *RhoA* and *DCRE* CRMs provide new insight into the *cis*-regulatory logic of the segment- and cell type-specific transcriptional responses, does this *cis*-regulatory logic provide insight into how Abd-A represses the *DCRE* and activates the *RhoA* CRMs? Currently, it is unclear how Abd-A/Pax2 complexes activate the *RhoA* CRM in sensory cells, but it is interesting to note that vertebrate Pax2 and Hox11 factors are thought to collaborate to activate the *six2* target gene and Hox11 contains an activation domain required for this function (Gong et al., 2007; Yallowitz et al., 2009). Moreover, the integration of Abd-A with either the Slp1/2 dFoxG factors or the En homeodomain factor provides a likely mechanism of repression as both dFoxG and En have been shown to recruit the well-established Groucho co-repressor protein (Jiménez et al., 1997; Andrioli et al., 2004). In addition, Agelopoulos et al. used a novel cell- and gene-specific ChIP strategy to demonstrate that while the regulatory element containing the *DCRE* loops and contacts the *Dll* promoter in thoracic segments, consistent with gene activation, the *DCRE* region does not contact the *Dll* promoter in the abdominal segments (Agelopoulos et al., 2012). Using whole embryo ChIP, the authors then found that the *Dll* enhancer region containing the *DCRE* was not only bound by Ubx and Abd-A but also was highly correlated with histone variant, H2Av. These data suggest that Ubx and/or Abd-A may recruit H2Av and result in decreased interactions between the *DCRE* and the *Dll* promoter (Agelopoulos et al., 2012). These data also further highlight how some Hox factors, most notably Ubx, can activate and repress gene expression by both increasing and decreasing chromatin accessibility in a gene-specific manner (Agelopoulos et al., 2012; Loker et al., 2021).

## Discussion

Several properties of the Hox factors make it particularly challenging to develop a general model that predicts the outcome of a Hox binding event to a regulatory element. First, Hox factors are expressed within many cell types of a segment, and yet most Hox target genes are regulated in only a small subset of the cells that express the Hox TF. Second, as mentioned throughout this review, all the members of the Hox TF family share a homeodomain that binds highly similar AT-rich sequences, and yet Hox TFs specify different segment identities and regional cell fates. Thus, we need to determine both the mechanisms that underlie how the same broadly expressed Hox TF can regulate target genes in a cell-type specific manner, and the mechanisms that underlie what makes each Hox TF different from each other to regulate the distinct combinations of target genes needed to specify different cell fates along the A-P axis.

This review summarized several studies that suggest select Hox factors can bind to inaccessible chromatin by intersecting genome binding and chromatin accessibility profiles (Beh et al., 2016; Porcelli et al., 2019; Bulajić et al., 2020). Moreover, at least in the case of Dfd and Abd-B, Hox TFs have the potential to increase chromatin accessibility even in the absence of nuclear Exd and Hth (Porcelli et al., 2019). However, intersecting genome binding and chromatin accessibility profiles only provides correlative evidence of pioneer-like activity, as it is possible that other TFs regulated by Dfd and Abd-B are ultimately the ones that open the chromatin. Thus, additional studies that combine the use of ATAC-seq, ChIP-seq, and Hox mutational analysis with genomic editing of known Hox binding sites will be required to confirm or refute Hox pioneer-like activity, much like the studies of Mariani et al. for PBX established pioneer activity in mouse epiblast stem cells (Mariani et al., 2021).

Through a combination of large-scale genomic, bioinformatics, and protein interactome approaches, the scientific field has recently identified numerous Hox-bound genomic elements as well as many Hox protein interactors that are likely to contribute to the diverse regulatory potential of Hox factors. Of particular note is that Hox TFs were found to form complexes with a surprisingly large number of other sequence specific TFs (Bischof et al., 2018; Carnesecchi et al., 2020). Taken together with the finding that several well-characterized Hox regulatory elements require TFBSs for additional TFs that are expressed in tissue- and cell type-restricted patterns, these results suggest that Hox-regulated CRMs function by integrating specific Hox TFs with numerous other TFs to yield accurate segment-, cell-, and gene-specific regulatory outcomes. The question that arises from these studies is do Hox TFs regulate each target gene by interactions with distinct combinations of TFs, and thus each Hox-regulated CRM will contain a relatively unique combination of TFBSs? Or do the Hox TFs regulate many different target genes *via* interactions with a common group of TFs such that potential *cis*-regulatory codes can be identified and used to predict Hox-regulated CRM output?

To answer these questions, future experiments will be needed to generate additional genomic binding data and transcriptomics data for Hox TFs as well as their potential partner proteins in defined cell types. For example, intersecting ChIP-seq for Hox TFs, Pbx/Exd, Hth/Meis, and other TFs from the same cell types would allow one to segregate Hox genomic binding events into distinct bins that are associated with the binding or lack thereof of additional TFs. Moreover, the use of higher resolution binding assays such as Cleavage Under Targets & Release Using Nuclease (CUT&RUN) or ChIP-Exonuclease can provide near bp resolution binding that reveals if adjacent sites are also occupied near the Hox TF binding site. Such an approach was recently utilized for the Gsx2 homeodomain TF to reveal distinct monomer versus dimer binding events using CUT&RUN assays and nucleotide footprinting analysis (Salomone et al., 2021). By combining high-resolution genomic binding data with transcriptomic studies using wild type and specific mutant cells (i.e., Hox mutant, Pbx/Exd mutant, etc), we will be better positioned to define which binding events are associated with gene expression changes. Lastly, bioinformatics can be used to perform unbiased searches for additional TF motifs as well as to search for potential constraints on the relationships between Hox TF sites and other TFBSs. Such an approach has already identified that many Hox genomic binding events enrich for coupled Pbx/Hox (vertebrates) or Exd/Hox (*Drosophila*) motifs even when the genomic binding assay was performed using a complex tissue composed of many cell types (Loker et al., 2021; Singh et al., 2021). These findings are consistent with the Pbx/Exd TFs serving as widespread Hox co-factor proteins in many tissues. Moreover, a recent study for HoxB1 combined genomic binding assays with transcriptomics and unbiased motif enrichment analysis to show that HoxB1 genomic binding events associated with gene repression, but not gene activation, are enriched for TFBSs for the REST transcriptional repressor (Singh et al., 2021). Hence, future studies focused on genomic binding assays for many Hox and other TFs in specific tissues will be needed to determine which TFs are likely to collaborate with specific Hox factors. Armed with the sequences of these potential regulatory elements, bioinformatics approaches will help to reveal if specific *cis*-regulatory codes underlie how Hox TFs are integrated with each different TF to regulate cell specific gene expression.

## References

[B1] Abbott M. K. Kaufman T. C. (1986). The Relationship between the Functional Complexity and the Molecular Organization of the Antennapedia Locus of *Drosophila melanogaster* . Genetics 114 (3), 919–942. 10.1093/genetics/114.3.919 3098627 PMC1203021

[B2] Agelopoulos M. McKay D. J. Mann R. S. (2012). Developmental Regulation of Chromatin Conformation by Hox Proteins in Drosophila. Cel Rep. 1 (4), 350–359. 10.1016/j.celrep.2012.03.003 PMC3329935 22523743

[B3] Andrioli L. P. Oberstein A. L. Corado M. S. G. Yu D. Small S. (2004). Groucho-dependent Repression by Sloppy-Paired 1 Differentially Positions Anterior Pair-Rule Stripes in the Drosophila Embryo. Develop. Biol. 276 (2), 541–551. 10.1016/j.ydbio.2004.09.025 15581884

[B4] Beh C. Y. El-Sharnouby S. Chatzipli A. Russell S. Choo S. W. White R. (2016). Roles of Cofactors and Chromatin Accessibility in Hox Protein Target Specificity. Epigenetics Chromatin 9 (1), 1–17. 10.1186/s13072-015-0049-x 26753000 PMC4705621

[B5] Bender W. Akam M. Karch F. Beachy P. A. Peifer M. Spierer P. (1983). Molecular Genetics of the Bithorax Complex in *Drosophila melanogaster* . Science 221 (4605), 23–29. 10.1126/science.221.4605.23 17737996

[B6] Berger M. F. Badis G. Gehrke A. R. Talukder S. Philippakis A. A. Peña-Castillo L. (2008). Variation in Homeodomain DNA Binding Revealed by High-Resolution Analysis of Sequence Preferences. Cell 133 (7), 1266–1276. 10.1016/j.cell.2008.05.024 18585359 PMC2531161

[B7] Bergstrom D. A. Penn B. H. Strand A. Perry R. L. S. Rudnicki M. A. Tapscott S. J. (2002). Promoter-specific Regulation of MyoD Binding and Signal Transduction Cooperate to Pattern Gene Expression. Mol. Cel 9 (3), 587–600. 10.1016/S1097-2765(02)00481-1 11931766

[B8] Berkes C. A. Bergstrom D. A. Penn B. H. Seaver K. J. Knoepfler P. S. Tapscott S. J. (2004). Pbx marks Genes for Activation by MyoD Indicating a Role for a Homeodomain Protein in Establishing Myogenic Potential. Mol. Cel 14 (4), 465–477. 10.1016/S1097-2765(04)00260-6 15149596

[B9] Biggin M. D. (2011). Animal Transcription Networks as Highly Connected, Quantitative Continua. Develop. Cel 21 (4), 611–626. 10.1016/j.devcel.2011.09.008 22014521

[B10] Bischof J. Duffraisse M. Furger E. Ajuria L. Giraud G. Vanderperre S. (2018). Generation of a Versatile BiFC ORFeome Library for Analyzing Protein-Protein Interactions in Live Drosophila. ELIFE 7, 1–24. 10.1101/34348310.7554/eLife.38853 PMC6177257 30247122

[B11] Brodu V. Elstob P. R. Gould A. P. (2002). Abdominal a Specifies One Cell Type in Drosophila by Regulating One Principal Target Gene. Development 129 (12), 2957–2963. 10.1242/dev.129.12.2957 12050142

[B12] Bulajić M. Srivastava D. Dasen J. S. Wichterle H. Mahony S. Mazzoni E. O. (2020). Differential Abilities to Engage Inaccessible Chromatin Diversify Vertebrate Hox Binding Patterns. Development 147, dev194761. 10.1242/dev.194761 33028607 PMC7710020

[B13] Bürglin T. R. Affolter M. (2016). Homeodomain Proteins: an Update. Chromosoma 125 (3), 497–521. 10.1007/s00412-015-0543-8 26464018 PMC4901127

[B14] Carnesecchi J. Sigismondo G. Domsch K. Baader C. E. P. Rafiee M. R. Krijgsveld J. (2020). Multi-level and Lineage-specific Interactomes of the Hox Transcription Factor Ubx Contribute to its Functional Specificity. Nat. Commun. 11, 1388. 10.1038/s41467-020-15223-x 32170121 PMC7069958

[B15] Carroll S. B. Weatherbee S. D. Langeland J. A. (1995). Homeotic Genes and the Regulation and Evolution of Insect wing Number. Nature 375 (6526), 58–61. 10.1038/375058a0 7723843

[B16] Casares F. Mann R. S. (1998). Control of Antennal versus Leg Development in Drosophila. Nature 392 (6677), 723–726. 10.1038/33706 9565034

[B17] Choe S.-K. Ladam F. Sagerström C. G. (2014). TALE Factors Poise Promoters for Activation by Hox Proteins. Develop. Cel 28 (2), 203–211. 10.1016/j.devcel.2013.12.011 PMC3930922 24480644

[B18] Choo S. W. Russell S. (2011). Genomic Approaches to Understanding Hox Gene Function. Adv. Genet. 76, 55. 10.1016/B978-0-12-386481-9.00003-1 22099692

[B19] Crocker J. Abe N. Rinaldi L. McGregor A. P. Frankel N. Wang S. (2015). Low Affinity Binding Site Clusters Confer HOX Specificity and Regulatory Robustness. Cell 160 (1–2), 191–203. 10.1016/j.cell.2014.11.041 25557079 PMC4449256

[B20] De Kumar B. Darland D. C. (2021). The Hox Protein Conundrum: The “Specifics” of DNA Binding for Hox Proteins and Their Partners. Develop. Biol. 477 (June), 284–292. 10.1016/j.ydbio.2021.06.002 34102167 PMC8846413

[B21] Desanlis I. Kherdjemil Y. Mayran A. Bouklouch Y. Gentile C. Sheth R. (2020). HOX13-dependent Chromatin Accessibility Underlies the Transition towards the Digit Development Program. Nat. Commun. 11, 2491. 10.1038/s41467-020-16317-2 32427842 PMC7237422

[B22] Duboule D. Dollé P. (1989). The Structural and Functional Organization of the Murine HOX Gene Family Resembles that of Drosophila Homeotic Genes. EMBO J. 8 (5), 1497–1505. 10.1002/j.1460-2075.1989.tb03534.x 2569969 PMC400980

[B23] Farnham P. J. (2009). Insights from Genomic Profiling of Transcription Factors. Nat. Rev. Genet. 10 (9), 605–616. 10.1038/nrg2636 19668247 PMC2846386

[B24] Fernandez Garcia M. Moore C. D. Schulz K. N. Alberto O. Donague G. Harrison M. M. (2019). Structural Features of Transcription Factors Associating with Nucleosome Binding. Mol. Cel 75 (5), 921–932. 10.1016/j.molcel.2019.06.009 PMC6731145 31303471

[B25] Fisher W. W. Li J. J. Hammonds A. S. Brown J. B. Pfeiffer B. D. Weiszmann R. (2012). DNA Regions Bound at Low Occupancy by Transcription Factors Do Not Drive Patterned Reporter Gene Expression in Drosophila. Proc. Natl. Acad. Sci. 109 (52), 21330–21335. 10.1073/pnas.1209589110 23236164 PMC3535648

[B26] Gebelein B. Culi J. Ryoo H. D. Zhang W. Mann R. S. (2002). Specificity of Distalless Repression and Limb Primordia Development by Abdominal Hox Proteins. Develop. Cel 3 (4), 487–498. 10.1016/S1534-5807(02)00257-5 12408801

[B27] Gebelein B. McKay D. J. Mann R. S. (2004). Direct Integration of Hox and Segmentation Gene Inputs during Drosophila Development. Nature 431 (7009), 653–659. 10.1038/nature02946 15470419

[B28] Gerber A. N. Klesert T. R. Bergstrom D. A. Tapscott S. J. (1997). Two Domains of MyoD Mediate Transcriptional Activation of Genes in Repressive Chromatin: A Mechanism for Lineage Determination in Myogenesis. Genes Develop. 11 (4), 436–450. 10.1101/gad.11.4.436 9042858

[B29] Giot L. Bader J. S. Brouwer C. Chaudhuri A. Kuang B. Li Y. (2003). A Protein Interaction Map of *Drosophila melanogaster* . Science 302 (5651), 1727–1736. 10.1126/science.1090289 14605208

[B30] Gong K.-Q. Yallowitz A. R. Sun H. Dressler G. R. Wellik D. M. (2007). A Hox-Eya-Pax Complex Regulates Early Kidney Developmental Gene Expression. Mol. Cel Biol. 27 (21), 7661–7668. 10.1128/mcb.00465-07 PMC2169072 17785448

[B31] Graham A. Papalopulu N. Krumlauf R. (1989). The Murine and Drosophila Homeobox Gene Complexes Have Common Features of Organization and Expression. Cell 57 (3), 367–378. 10.1016/0092-8674(89)90912-4 2566383

[B32] Hajirnis N. Mishra R. K. (2021). Homeotic Genes: Clustering, Modularity, and Diversity. Front. Cel Develop. Biol. 9 (August), 1–20. 10.3389/fcell.2021.718308 PMC8386295 34458272

[B33] Hueber S. D. Weiller G. F. Djordjevic M. A. Frickey T. (2010). Improving Hox Protein Classification across the Major Model Organisms. PLoS ONE 5, e10820. 10.1371/journal.pone.0010820 20520839 PMC2876039

[B34] Iwafuchi-Doi M. Zaret K. S. (2014). Pioneer Transcription Factors in Cell Reprogramming. Genes Dev. 28 (24), 2679–2692. 10.1101/gad.253443.114 25512556 PMC4265672

[B35] Jiménez G. Paroush Z. e. Ish-Horowicz D. (1997). Groucho Acts as a Corepressor for a Subset of Negative Regulators, Including Hairy and Engrailed. Genes Develop. 11 (22), 3072–3082. 10.1101/gad.11.22.3072 9367988 PMC316696

[B36] Jolma A. Yan J. Whitington T. Toivonen J. Nitta K. R. Rastas P. (2013). DNA-binding Specificities of Human Transcription Factors. Cell 152 (1–2), 327–339. 10.1016/j.cell.2012.12.009 23332764

[B37] Kaufman T. C. Lewis R. Wakimoto B. (1980). Cytogenetic Analysis of Chromosome 3 in *Drosophila melanogaster*: The Homoeotic Gene Complex in Polytene Chromosome Interval 84A-B. Genetics 94 (1), 115–133. 10.1093/genetics/94.1.115 17248988 PMC1214128

[B38] Kribelbauer J. F. Rastogi C. Bussemaker H. J. Mann R. S. (2019). Low-affinity Binding Sites and the Transcription Factor Specificity Paradox in Eukaryotes. Annu. Rev. Cel Dev. Biol. 35, 357–379. 10.1146/annurev-cellbio-100617-062719 PMC6787930 31283382

[B39] Lambert B. Vandeputte J. Remacle S. Bergiers I. Simonis N. Twizere J. C. (2012). Protein Interactions of the Transcription Factor Hoxa1. BMC Dev. Biol. 12, 29. 10.1186/1471-213X-12-29 23088713 PMC3514159

[B40] LaRonde-LeBlanc N. A. Wolberger C. (2003). Structure of HoxA9 and Pbx1 Bound to DNA: Hox Hexapeptide and DNA Recognition Anterior to Posterior. Genes Develop. 17 (16), 2060–2072. 10.1101/gad.1103303 12923056 PMC196259

[B41] Lewis E. B. (1978). A Gene Complex Controlling Segmentation in Drosophila. Nature 276 (5688), 565–570. 10.1038/276565a0 103000

[B42] Li X. Murre C. McGinnis W. (1999). Activity Regulation of a Hox Protein and a Role for the Homeodomain in Inhibiting Transcriptional Activation. EMBO J. 18 (1), 198–211. 10.1093/emboj/18.1.198 9878063 PMC1171115

[B43] Li-Kroeger D. Cook T. A. Gebelein B. (2012). Integration of an Abdominal Hox Complex with PAX2 Yields Cell-specific EGF Secretion from Drosophila Sensory Precursor Cells. Development 139 (9), 1611–1619. 10.1242/dev.077842 22438572 PMC3317967

[B44] Li-Kroeger D. Witt L. M. Grimes H. L. Cook T. A. Gebelein B. (2008). Hox and Senseless Antagonism Functions as a Molecular Switch to Regulate EGF Secretion in the Drosophila PNS. Develop. Cel 15 (2), 298–308. 10.1016/j.devcel.2008.06.001 PMC2610489 18694568

[B45] Loker R. Sanner J. E. Mann R. S. (2021). Cell-type-specific Hox Regulatory Strategies Orchestrate Tissue Identity. Curr. Biol. 31 (19), 4246–4255. 10.1016/j.cub.2021.07.030 34358443 PMC8511240

[B46] Luck K. Kim D. K. Lambourne L. Spirohn K. Begg B. E. Bian W. (2020). A Reference Map of the Human Binary Protein Interactome. Nature 580 (7803), 402–408. 10.1038/s41586-020-2188-x 32296183 PMC7169983

[B47] Luo Z. Rhie S. K. Farnham P. J. (2019). The Enigmatic Hox Genes: Can We Crack Their Code? Cancers 11, 323. 10.3390/cancers11030323 PMC6468460 30866492

[B48] Maeda R. K. Karch F. (2009). The Bithorax Complex of Drosophila. Curr. Top. Develop. Biol. 88 (09), 1–33. 10.1016/S0070-2153(09)88001-0 19651300

[B49] Mann R. S. Affolter M. (1998). Hox Proteins Meet More Partners. Curr. Opin. Genet. Develop. 8 (4), 423–429. 10.1016/S0959-437X(98)80113-5 9729718

[B50] Mann R. S. Lelli K. M. Joshi R. (2009). Hox Specificity Unique Roles for Cofactors and Collaborators. Curr. Top. Develop. Biol. 88, 63. 10.1016/S0070-2153(09)88003-4 PMC2810641 19651302

[B51] Mann R. S. (1997). Why areHox Genes Clustered? BioEssays 19 (8), 661–664. 10.1002/bies.950190804 9264246

[B52] Mariani L. Guo X. Menezes N. A. Drozd A. M. Çakal S. D. Wang Q. (2021). A TALE/HOX Code Unlocks WNT Signalling Response towards Paraxial Mesoderm. Nat. Commun. 12 (1), 5136–5219. 10.1038/s41467-021-25370-4 34446717 PMC8390530

[B53] Mark M. Rijli F. M. Chambon P. (1997). Homeobox Genes in Embryogenesis and Pathogenesis. Pediatr. Res. 42 (4), 421–429. 10.1203/00006450-199710000-00001 9380431

[B54] McGinnis W. Garber R. L. Wirz J. Kuroiwa A. Gehring W. J. (1984a). A Homologous Protein-Coding Sequence in drosophila Homeotic Genes and its Conservation in Other Metazoans. Cell 37 (2), 403–408. 10.1016/0092-8674(84)90370-2 6327065

[B55] McGinnis W. Levine M. S. Hafen E. Kuroiwa A. Gehring W. J. (1984b). A Conserved DNA Sequence in Homoeotic Genes of the Drosophila Antennapedia and Bithorax Complexes. Nature 308 (5958), 428–433. 10.1038/308428a0 6323992

[B56] McKay D. J. Lieb J. D. (2013). A Common Set of DNA Regulatory Elements Shapes Drosophila Appendages. Develop. Cel 27 (3), 306–318. 10.1016/j.devcel.2013.10.009 PMC3866527 24229644

[B57] Merabet S. Litim-Mecheri I. Karlsson D. Dixit R. Saadaoui M. Monier B. (2011). Insights into Hox Protein Function from a Large Scale Combinatorial Analysis of Protein Domains. Plos Genet. 7, e1002302. 10.1371/journal.pgen.1002302 22046139 PMC3203194

[B58] Merabet S. Mann R. S. (2016). To Be Specific or Not: The Critical Relationship between Hox and TALE Proteins. Trends Genet. 32 (6), 334–347. 10.1016/j.tig.2016.03.004 27066866 PMC4875764

[B59] Moens C. B. Selleri L. (2006). Hox Cofactors in Vertebrate Development. Develop. Biol. 291 (2), 193–206. 10.1016/j.ydbio.2005.10.032 16515781

[B60] Morata G. Macías A. Urquía N. González-Reyes A. (1990). Homoeotic Genes. Semin. Cel Biol. 1 (3), 219–227. 1983316

[B61] Noordermeer D. Duboule D. (2013). Chromatin Architectures and Hox Gene Collinearity. Curr. Top. Develop. Biol. 104, 113. 10.1016/B978-0-12-416027-9.00004-8 23587240

[B62] Pearson J. C. Lemons D. McGinnis W. (2005). Modulating Hox Gene Functions during Animal Body Patterning. Nat. Rev. Genet. 6 (12), 893–904. 10.1038/nrg1726 16341070

[B63] Porcelli D. Fischer B. Russell S. White R. (2019). Chromatin Accessibility Plays a Key Role in Selective Targeting of Hox Proteins. Genome Biol. 20 (1), 115–119. 10.1186/s13059-019-1721-4 31159833 PMC6547607

[B64] Quinonez S. C. Innis J. W. (2014). Human HOX Gene Disorders. Mol. Genet. Metab. 111 (1), 4–15. 10.1016/j.ymgme.2013.10.012 24239177

[B65] Rambaldi I. Kovàcs E. N. Featherstone M. S. (1994). A Proline-Rich Transcriptional Activation Domain in Murine HOXD-4 (HOX-4.2). Nucl. Acids Res. 22 (3), 376–382. 10.1093/nar/22.3.376 7907418 PMC523592

[B66] Rolland T. Taşan M. Charloteaux B. Pevzner S. J. Zhong Q. Sahni N. (2014). A Proteome-Scale Map of the Human Interactome Network. Cell 159 (5), 1212–1226. 10.1016/j.cell.2014.10.050 25416956 PMC4266588

[B67] Rual J.-F. Venkatesan K. Hao T. Hirozane-Kishikawa T. Dricot A. Li N. (2005). Towards a Proteome-Scale Map of the Human Protein-Protein Interaction Network. Nature 437 (7062), 1173–1178. 10.1038/nature04209 16189514

[B68] Sagerström C. G. (2004). pbX Marks the Spot. Develop. Cel 6, 737–738. 10.1016/j.devcel.2004.05.015 15177017

[B69] Salomone J. Qin S. Fufa T. D. Cain B. Farrow E. Guan B. (2021). Conserved Gsx2/Ind Homeodomain Monomer versus Homodimer DNA Binding Defines Regulatory Outcomes in Flies and Mice. Genes Dev. 35 (1), 157–174. 10.1101/GAD.343053.120 33334823 PMC7778271

[B70] Schnabel C. A. Abate-Shen C. (1996). Repression by HoxA7 Is Mediated by the Homeodomain and the Modulatory Action of its N-Terminal-Arm Residues. Mol. Cel Biol. 16 (6), 2678–2688. 10.1128/mcb.16.6.2678 PMC231258 8649375

[B71] Schneuwly S. Klemenz R. Gehring W. J. (1987). Redesigning the Body Plan of Drosophila by Ectopic Expression of the Homoeotic Gene Antennapedia. Nature 325 (6107), 816–818. 10.1038/325816a0 3821869

[B72] Shokri L. Inukai S. Hafner A. Weinand K. Hens K. Vedenko A. (2019). A Comprehensive *Drosophila melanogaster* Transcription Factor Interactome. Cel Rep. 27 (3), 955–970. 10.1016/j.celrep.2019.03.071 PMC6485956 30995488

[B73] Singh N. P. De Kumar B. Paulson A. Parrish M. E. Scott C. Zhang Y. (2021). Genome-wide Binding Analyses of HOXB1 Revealed a Novel DNA Binding Motif Associated with Gene Repression. J. Dev. Biol. 9 (1), 1–20. 10.3390/JDB9010006 33546292 PMC7931043

[B74] Singh N. P. de Kumar B. Paulson A. Parrish M. E. Zhang Y. Florens L. (2020). A Six-Amino-Acid Motif Is a Major Determinant in Functional Evolution of HOX1 Proteins. Genes Dev. 34 (23–24), 1680–1696. 10.1101/gad.342329.120 33184220 PMC7706710

[B75] Slattery M. Riley T. Liu P. Abe N. Gomez-Alcala P. Dror I. (2011). Cofactor Binding Evokes Latent Differences in DNA Binding Specificity between Hox Proteins. Cell 147 (6), 1270–1282. 10.1016/j.cell.2011.10.053 22153072 PMC3319069

[B76] Stanyon C. A. Liu G. Mangiola B. A. Patel N. Giot L. Kuang B. (2004). A Drosophila Protein-Interaction Map Centered on Cell-Cycle Regulators. Genome Biol. 5, R96. 10.1186/gb-2004-5-12-r96 15575970 PMC545799

[B77] Tycko J. DelRosso N. Hess G. T. Banerjee A. Van M. V. Ego B. K. (2020). High-Throughput Discovery and Characterization of Human Transcriptional Effectors. Cell 183 (7), 2020–2035. 10.1016/j.cell.2020.11.024 33326746 PMC8178797

[B78] Uhl J. D. Cook T. A. Gebelein B. (2010). Comparing Anterior and Posterior Hox Complex Formation Reveals Guidelines for Predicting Cis-Regulatory Elements. Dev. Biol. 343 (1–2), 154–166. 10.1016/j.ydbio.2010.04.004 20398649 PMC2885469

[B79] Uhl J. D. Zandvakili A. Gebelein B. (2016). A Hox Transcription Factor Collective Binds a Highly Conserved Distal-Less Cis-Regulatory Module to Generate Robust Transcriptional Outcomes. Plos Genet. 12 (4), e1005981. 10.1371/journal.pgen.1005981 27058369 PMC4825978

[B80] Vachon G. Cohen B. Pfeifle C. McGuffin M. E. Botas J. Cohen S. M. (1992). Homeotic Genes of the Bithorax Complex Repress Limb Development in the Abdomen of the Drosophila Embryo through the Target Gene Distal-Less. Cell 71 (3), 437–450. 10.1016/0092-8674(92)90513-C 1358457

[B81] Walhout A. J. (2011). What Does Biologically Meaningful Mean? A Perspective on Gene Regulatory Network Validation. Genome Biol. 12 (4), 109–117. 10.1186/gb-2011-12-4-109 21489330 PMC3218850

[B82] Walter J. Dever C. A. Biggin M. D. (1994). Two Homeo Domain Proteins Bind with Similar Specificity to a Wide Range of DNA Sites in Drosophila Embryos. Genes Develop. 8 (14), 1678–1692. 10.1101/gad.8.14.1678 7958848

[B83] Yallowitz A. R. Gong K.-Q. Swinehart I. T. Nelson L. T. Wellik D. M. (2009). Non-homeodomain Regions of Hox Proteins Mediate Activation versus Repression of Six2 via a Single Enhancer Site *In Vivo* . Develop. Biol. 335 (1), 156–165. 10.1016/j.ydbio.2009.08.020 19716816 PMC2791332

[B84] Yu H. Tardivo L. Tam S. Weiner E. Gebreab F. Fan C. (2011). Next-generation Sequencing to Generate Interactome Datasets. Nat. Methods 8 (6), 478–480. 10.1038/nmeth.1597 21516116 PMC3188388

[B85] Zandvakili A. Campbell I. Gutzwiller L. M. Weirauch M. T. Gebelein B. (2018). Degenerate Pax2 and Senseless Binding Motifs Improve Detection of Low-Affinity Sites Required for Enhancer Specificity. Plos Genet. 14 (4), e1007289–25. 10.1371/journal.pgen.1007289 29617378 PMC5902045

[B86] Zandvakili A. Gebelein B. (2016). Mechanisms of Specificity for Hox Factor Activity. J. Dev. Biol. 4, 16. 10.3390/jdb4020016 27583210 PMC5003318

[B87] Zandvakili A. Uhl J. D. Campbell I. Salomone J. Song Y. C. Gebelein B. (2019). The Cis-Regulatory Logic Underlying Abdominal Hox-Mediated Repression versus Activation of Regulatory Elements in Drosophila. Develop. Biol. 445 (2), 226–236. 10.1016/j.ydbio.2018.11.006 30468713 PMC6333500

[B88] Zaret K. S. (2020). Pioneer Transcription Factors Initiating Gene Network Changes. Annu. Rev. Genet. 54, 367–385. 10.1146/annurev-genet-030220-015007 32886547 PMC7900943

[B89] Zeiske T. Baburajendran N. Kaczynska A. Brasch J. Palmer A. G. Shapiro L. (2018). Intrinsic DNA Shape Accounts for Affinity Differences between Hox-Cofactor Binding Sites. Cel Rep. 24 (9), 2221–2230. 10.1016/j.celrep.2018.07.100 PMC6166240 30157419

[B90] Zhu F. Farnung L. Kaasinen E. Sahu B. Yin Y. Wei B. (2018). The Interaction Landscape between Transcription Factors and the Nucleosome. Nature 562 (7725), 76–81. 10.1038/s41586-018-0549-5 30250250 PMC6173309

